# Exosomal MiR‐500a‐3p promotes cisplatin resistance and stemness via negatively regulating FBXW7 in gastric cancer

**DOI:** 10.1111/jcmm.15524

**Published:** 2020-06-25

**Authors:** Hao Lin, Liang Zhang, Caihua Zhang, Pengpeng Liu

**Affiliations:** ^1^ Department of General Surgery XuZhou Central Hospital Xuzhou China

**Keywords:** chemoresistance, cisplatin, exosome, gastric cancer, miRNA‐500a‐3p

## Abstract

Chemoresistance has been a major challenge in advanced gastric cancer (GC) therapy. Exosomal transfer of oncogenic miRNAs implicates important effects in mediating recipient cell chemoresistance by transmitting active molecules. In this study, we found that microRNA‐500a‐3p was highly expressed in cisplatin (DDP) resistant GC cells (MGC803/DDP and MKN45/DDP) and their secreted exosomes than that in the corresponding parental cells. MGC803/DDP‐derived exosomes enhance DDP resistance and stemness properties of MGC803 recipient cells via exosomal delivery of miR‐500a‐3p in vitro and in vivo through targeting FBXW7. However, reintroduction of FBXW7 in MGC803 cells reverses miR‐500a‐3p‐mediated DDP resistance as well as stemness properties. Furthermore, elevated miR‐500a‐3p in the plasma exosomes of GC patients is correlated with DDP resistance and thereby results in poor progression‐free prognosis. Our finding highlights the potential of exosomal miR‐500a‐3p as an potential modality for the prediction and treatment of GC with chemoresistance.

## 1 INTRODUCTION

Gastric cancer (GC) is a serious global public health problem that rank the sixth most common malignancy and the third leading cause of cancer‐related deaths over the world.^1^ Because of a majority of GC patients diagnosed in advanced or metastatic stages,^2^ chemotherapy has been the pivotal therapeutic strategy to improve prognosis.^3^ In this regard, cisplatin (DDP) is one of the most important and basic drugs in chemotherapy regimen against advanced GC.^4^ However, chemoresistance—whether intrinsic or acquired—remains an inevitable obstacle in most GC patients and represents the most important cause of recurrence and mortality in GC.^5^

Exosomes have been identified as an important group of 30‐100 nm sized extracellular vesicles with lipid membranes and cup‐shaped constructio.^6^ When endosomal multivesicular bodies merge into cell membrane, exosomes containing biomolecules can be discharged into the extracellular surroundings.^7^, ^8^, ^9^ Later, exosomes that uptake by surrounding or distant recipient cells would carry out various biological activities such as immunomodulation,^10^ angiogenesis,^11^ autophagy,^12^ stem cell differentiation^13^ and intercellular communication.^14^ While in cancer research, a plethora of recent evidence shows exosomes participate in tumour microenvironment remodelling, development, metastasis and chemoresistance through initiating or suppressing various signalling pathways in the recipient cells.^15^ RNA cargo that protected by exosomes from digestion has garnered much attention from researchers, especially microRNAs (miRNAs). MiRNAs are a class of 18‐22 nucleotides small single‐stranded non‐coding RNA molecules that promote mRNA cleavage and subsequent degradation by binding to the complementary 3′ untranslated region (UTR) of the mRNA and thereby regulate protein regulation.^16^ Emerging evidence demonstrated that exosomal miRNAs from cancer cells played important roles in mediating tumour growth, metastasis and chemoresistance.^17^, ^18^, ^19^, ^20^ However, the mechanisms of exosomeal miRNAs in DDP resistant GC are still waiting for exposure.

In this study, the effects of exosomal miRNAs on DDP resistance in GC cells were investigated. Furthermore, we identify exosomal miR‐500a‐3p promote DDP resistance and CSCs properties in GC cells by down‐regulating FBXW7. Clinically, miR‐500a‐3p expression correlated positively with DDP resistance as well as recurrence and might be a potential therapeutic predictor of DDP‐based chemotherapy in GC patients.

## 2 MATERIALS AND METHODS

### 2.1 Cell culture

GC cell lines MGC803 and MKN45 were obtained from the ATCC. They have been authenticated by a STR (Short TandemRepeat) DNA profiling. MGC803 and MKN45 lines cultured in RPMI 1640 (HyClone, Logan, UT, USA) with 10% foetal bovine serum (FBS; Thermo Fisher Scientific, Waltham, MA, USA), penicillin (100 U/mL) and streptomycin (100 mg/mL) (Invitrogen, Carlsbad, CA, USA). Successive exposure to elevating concentrations of DDP (Sigma‐Aldrich, St. Louis, MO, USA) was used to establish DDP resistant MGC803 (MGC803/DDP) and MKN45 (MKN45/DDP) cells. All cells were grown in a moist atmosphere with 5% CO_2_ incubator at 37°C.

### 2.2 Patient samples and ethical statement

Plasma samples were collected from 55 III stage GC patients receiving DDP‐based chemotherapy after gastrectomy with D2 lymphadenectomy at XuZhou Central Hospital. The patients were designated into the chemoresistant group (n = 25) and chemosensitive group (n = 30) according to the RECIST 1.1 (Response Evaluation Criteria in Solid Tumors). This research was performed with approval of Research Ethics Committee of XuZhou Central Hospital. All participants gave written informed consent before blood sample collection.

### 2.3 Exosome isolation and purification

An ExoQuick precipitation kit (System Biosciences, LLC, Palo Alto, CA, USA) was used to extract and purify exosomes in accordance with the manufacturer's instruction. Briefly, the culture medium or plasma was harvested and centrifuged at 3000 *g* for 15 minutes. Then, the obtained supernatant was mixed with ExoQuick precipitation solution and incubated at 4°C for 30 minutes, centrifuged at 1500 *g* for 25 minutes. After removing the supernatant, the exosome pellets were centrifuged for another 10 minutes at 1500 *g* to discard the extra liquid. Finally, the exosomes were conserved in PBS.

### 2.4 Characterization of exosomes

The morphology of exosome was observed by transmission electron microscopy. Briefly, exosomes were fixed by 1% glutaraldehyde and incubated at 4°C. Next, 10 μL of the medium was placed onto formvar/carboncoated copper grids, followed by dyeing with 3% aqueous phosphotungstic aid for 35 seconds. Subsequently, exosomes were observed with a transmission electron microscopy (Tecnai 12; Philips, Amsterdam, Netherlands). Size distribution of exosomes was analysed by NanoSight LM10 system which was equipped with a fast video capture and particle‐tracking software (NanoSight, Amesbury, UK). Western blot analysis was performed to detect exosome markers CD63 and CD81.

### 2.5 Exosomes and miR‐500a‐3p internalization assays

Exosomes were labelled with PKH‐67 green fluorescent Cell Linker Kit (Sigma‐Aldrich, USA) according to the manufacturer's protocol. The labelled exosomes were co‐cultured with MGC803 cells for 30 hours at 37°C. For the transfer of exosomal miR‐500a‐3p, PKH‐67 labelled miR‐500a‐3p was transfected to MGC803 cells by liposome 2000 (Invitrogen). The PKH‐67‐miR‐301a‐expressing MGC803 cells were grown on the 0.4 mm pore size transwell (Thermo Fisher Scientific), and then co‐cultured with MGC803 cells that had been grown on the cover slips in the bottom well of the transwell for 30 hours. The uptake of labelled exosomes or miR‐500a‐3p by the recipient MGC803 cells was observed using a Nikon Eclipse fluorescence microscope (Nikon, Tokyo, Japan).

### 2.6 Cell proliferation assay

Cell Counting Kit‐8 (CCK‐8; Sangon Biotech, Shanghai, China) was used to observe cell viability. Briley, GC cells were seeded into 96‐well plates and exposed to different concentration of DDP for 30 hours. Subsequently, cell viability was examined by CCK‐8 following the manufacture's specification. Finally, the absorbance was read under a microplate reader (Bio‐Rad, Hercules, CA, USA) at 450 nm. IC_50_ values were calculated on the basis of the charted dose‐response curve from GraphPad Prism 8.0 software (GraphPad Software, Inc., San Diego, CA, USA).

### 2.7 Immunofluorescence assay

Transfected or exosomes‐treated GC cells were fixed in 4% paraformaldehyde for 10 minutes, blocked with PBS buffer containing 5% bovine serum albumin. Then, those cells incubated with antibodies at 4°C overnight, followed by incubation with fluorescein isothiocyanate (FITC)‐conjugated secondary antibody and the nuclear counterstain diaminophenylindole (DAPI). After rinsing, the cells were analysed using immunofluorescence microscopy.

### 2.8 Sphere formation assay

Transfected or exosomes‐treated 600 GC cells were seeded in ultra‐low‐attachment 24‐well plates (Corning Life Sciences, Corning, NY, USA) with 0.8% methyl cellulose (Sigma, St. Louis, MO, USA) supplemented with 20 μL/mL B27 supplement (Life Technologies, Carlsbad, CA, USA), 20 ng/mL basic fibroblast growth factor (bFGF; Gibco, Rockville, MD, USA), 10 ng/mL EGF (Gibco), LIF (Gibco), 1% l‐glutamine (Gibco) and 1% penicillin‐streptomycin sulphate (Thermo Fisher Scientific) for 2 weeks. The number of sphere in each well ≥50 μm in diameter was counted under a microscope. Sphere formation rate for each well was the ratio of colony number to total cell number per well.

### 2.9 Western blot assay

Proteins were extracted with a lysis buffer and then quantified by a bicinchoninic acid protein assay. Equivalent amounts of cell lysates were separated using SDS‐PAGE and transferred to a polyvinylidene difluoride membrane (Roche Applied Sciences, Indianapolis, IA, USA). Membranes were immunoblotted overnight at 4°C with corresponding antibodies (Table S1). The bands were visualized using Pierce ECL Western Blotting Substrate (Thermo Fisher Scientific). Image density of the immunoblotting was determined by Gel densitometry (Bio‐Rad).

### 2.10 RNA extraction and real‐time qRT‐PCR

Total RNA for cultured cells and exosomes were extracted with using Trizol Reagent (Takara Bio, Inc., Shiga, Japan). The mRNA expressions were detected by the PrimeScript RT Reagent Kit and SYBR Premix Ex Taq (Takara Bio, Inc.). GAPDH was used as control. All the primers designed for qPCR were listed in Table S1. All‐in‐One microRNA qRT‐PCR Detection Kits (GeneCopoeia, Inc., Rockville, MD, USA) were used to detect miRNA expression and U6 used as a control. Every experiment was repeated three times according to the manufacturer's protocol. Final data were analysed with the $2^{-\Delta \Delta C_{t}}$ method.

### 2.11 Luciferase assays

For luciferase reporter assays, the 293T cells were cotransfected with wild‐type or mutant FBXW7 3′UTR psiCHECK‐2 plasmid (Promega, Madison, WI, USA) and mimic‐miR‐500a‐3p or anti–miR‐500a‐3p (Ribo, Guangzhou, China) or control using Lipofectamine 2000 (Invitrogen). Luciferase activity was measured 48 hours after transfection by the dual‐luciferase reporter assay system (Promega, Madison, USA). Firefly luciferase signal was used for normalization. Each assay was repeated in three independent experiments.

### 2.12 Plasmid construction and RNA transfection

Mimic, anti–miRNA‐500a‐3p, scrambled control was obtained from GenePharma and was transfected at a final concentration of 100 nmol/L. For FBXW7 overexpression, PCR‐amplified full‐length human FBXW7 cDNA was cloned into pcDNA3.1 (pcDNA3.1‐ FBXW7) and transfected to GC cells via Lipofectamine 2000 (Invitrogen; Thermo Fisher Scientific, Inc.) as the delivery agent, according to the manufacturer's protocol.

### 2.13 Abdominal tumorigenicity assay in vivo

All animal experiments were conducted in accordance with the principles and procedures approved by the Committee on the Ethics of XuZhou Central Hospital. In BALB/c nude mice model, 5 × 10^6^ GC cells, including DDP resistant or not or FBXW7‐expression plasmid transfected, were injected into the abdominal cavity for tumorigenicity (n = 5 in each group) and then indicated treatment such as PBS, exosomes or conditioned medium (CM) without exosomes would be injected into abdominal cavity every 5 days. Meanwhile, all mice were administrated DDP (2 mg/kg) by abdominal injection after above treatment. Tumour growth was monitored and quantified using an IVIS‐100 system (Caliper Life Sciences, Boston, MA, USA) every 5 days. Twenty days later, all mice were killed after luciferase signal intensity examination and the xenograft tumour were subjected to H&E staining.

### 2.14 Statistics

All in vitro experiments were repeated at least in triplicate. The data were represented as either a scatter plots or bar graphs with means ± standard error deviation of the mean (SEM). The statistical analysis was performed using SPSS software (version 13.0, New York, NY, USA). Statistical significance between two groups was determined using a two‐tailed Student's *t* test. To compare multiple groups, one‐way analysis of variance (ANOVA) followed by a Bonferroni‐Dunn test was performed. The GC patients were divided into high expression group and low expression group according to the median ofmiR‐500a‐3p expression and Kaplan‐Meier survival analysis was implemented to compare GC patient progression‐free survival by log‐rank test. The receiver operating characteristic (ROC) curve was applied to determine the area under the curve (AUC) values for exosomal miR‐500a‐3p in plasma by the GraphPad Prism software (GraphPad Software, Inc.). *P* < 0.05 was considered statistically significant.

## 3 RESULT

### 3.1 DDP resistant GC cells exhibited higher tumorigenesis and CSCs properties

**FIGURE 1:**
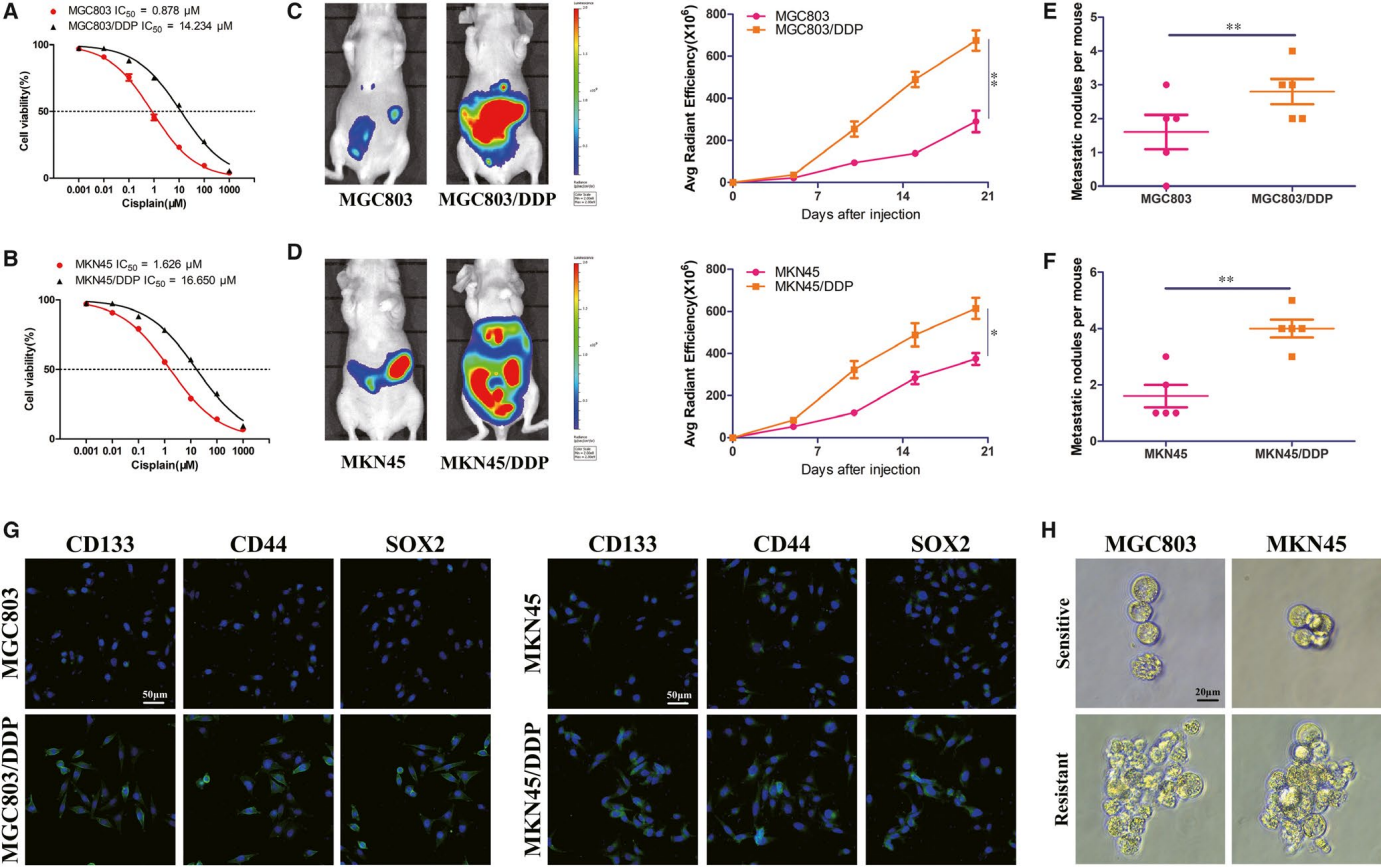
DDP resistant MGC803 and MKN45 cells display stem cell‐like features. A, B, Proliferation assay analysis and IC_50_ value of DDP resistant GC cells (MGC803/DDP and MKN45/DDP) and corresponding parental cells (MGC803 and MKN45). C, D, Representative images showed the bioluminescent signals of abdominal tumorigenesis by indicated GC cells under DDP treatment (left panel). Comparison of bioluminescent signals on the day 21 was analysed (right panel), five nude mice in each group. E, F, Comparison of abdominal metastatic nodules by indicated GC cells under DDP treatment. G, Expression level of stemness markers CD133, CD44 and SOX2 in indicated GC cells by confocal microscopy. H, Sphere formation assay in indicated GC cells. *P < 0.05, **P < 0.01

To explore the underlying molecular mechanism of GC DDP resistance, DDP‐resistant cell lines, MGC803/DDP and MKN45/DDP, were established by treating MGC803 and MKN45 cells with gradually elevating concentrations of DDP in sequential passages. First of all, we examined the cell viability and IC_50_ values in MGC803, MGC803/DDP and MKN45, MKN45/DDP by exposing them to different concentrations of DDP for 30 hours. As shown in Figure 1A,B, compared to MGC803 and MKN45 cells, MGC803/DDP and MKN45/DDP presented higher cell viability and IC_50_ value. Furthermore, both MGC803/DDP (Figure 1C,E) and MKN45/DDP (Figure 1D,F) exhibited enhanced abdominal tumorigenesis and more metastatic nodules compared with their corresponding parental cells. Accumulating evidence demonstrates that CSCs play important roles in chemoresistance of many human tumours. In our established DDP resistant GC cells lines, higher proportion of CSCs markers CD133+, CD44+ and SOX+ were observed in MGC803/DDP and MKN45/DDP cells (Figure 1G). Consistently, MGC803/DDP and MKN45/DDP cells could formed larger spheres compared with those sensitive cells (Figure 1H). These data suggested that DDP resistant GC cells were successfully established and those DDP resistant GC cells exhibited higher tumorigenesis and CSCs properties.

### 3.2 MGC803/DDP‐derived exosomes conferred DDP resistance and promote CSCs properties in recipient MGC803 cells

**FIGURE 2:**
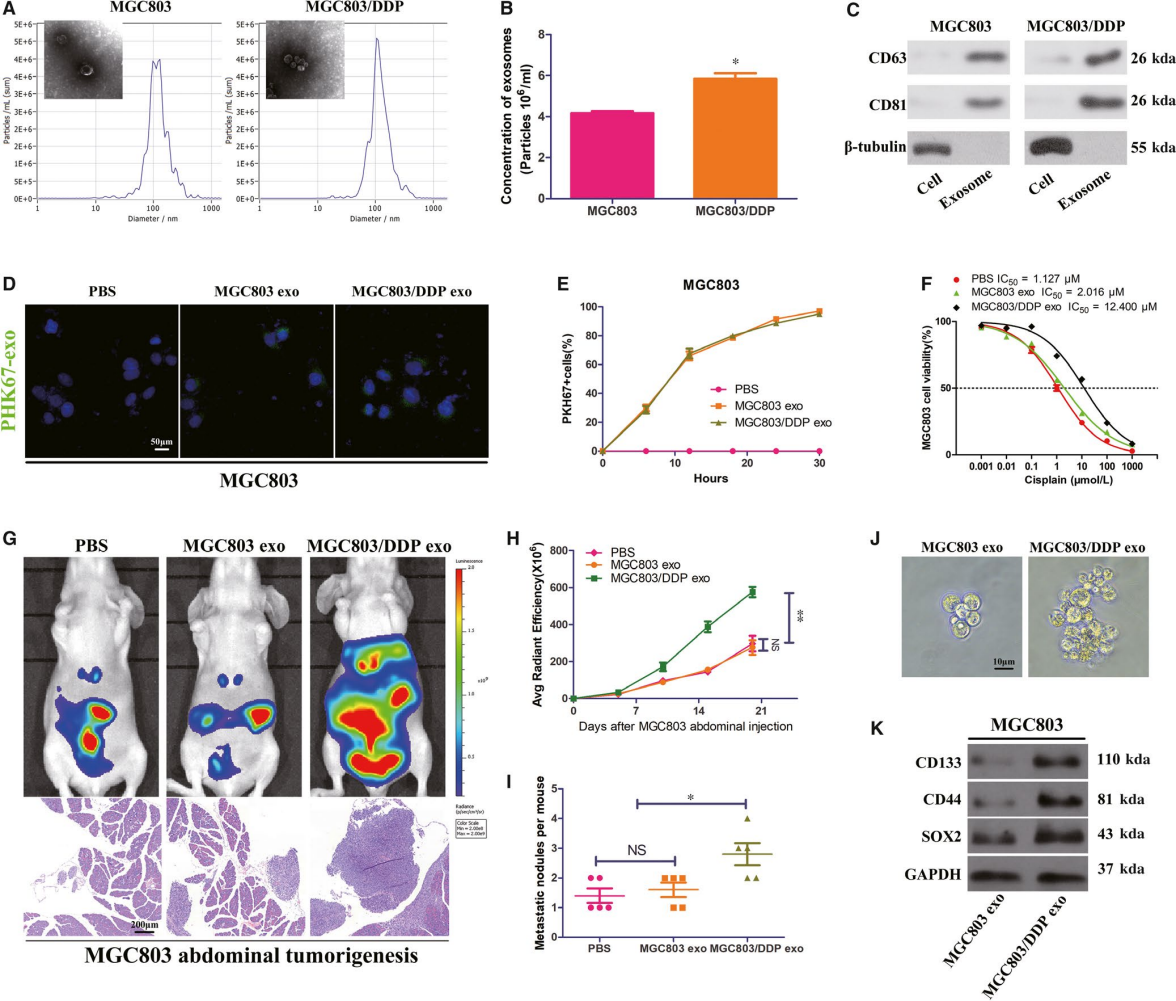
MGC803/DDP‐derived exosomes disseminate DDP resistance and promote CSCs properties in recipient MGC803 cells. A, Electron microscopy and NTA images of exosomes isolated from MGC803 and MGC803/DDP cells medium. B, C, Identical quantity of MGC803 and MGC803/DDP cells were cultured 30 h and then their exosomes concentrations and positive markers (CD63, HSP81) were analysed by NTA (B) and Western blot (C). D, MGC803 cells were incubated with PHK67‐labelled exosomes from MGC803 and MGC803/DDP cells for 30 h, and the internalization of exosomes was detected by confocal microscopy. E, Uptake efficiency of PHK67‐labelled exosomes was examined by confocal microscopy. F, Proliferation assay analysis and IC_50_ value of MGC803 cells co‐cultured with PBS, MGC830 exosomes or MGC803/DDP exosomes under DDP treatment. G, Representative bioluminescent images and microscopy observations showed effects of PBS, MGC803 exosomes or MGC803/DDP exosomes on abdominal tumorigenesis under DDP treatment. H, I, Comparison of bioluminescent signals and abdominal metastatic nodules after indicated treatment, five nude mice in each group. J, K, Effect of MGC803 exosomes and MGC803/DDP exosomes on sphere formation (J) and expression levels of stemness markers (K) in MCG803 cells by Western blot. *P < 0.05, **P < 0.01

Recent studies indicated that exosomes derived from cancer cells were implicated in chemotherapy resistance.^19^, ^20^ We speculated that exosomes from DDP resistant GC cells might generate their effects on recipient cells. In order to verify this hypothesis, we isolated exosomes from the conditioned medium (CM) of MGC803 and MGC803/DDP cells. Transmission electron microscopy (TEM) revealed a cup‐shaped vesicles with bilayered membranes and the Nanosight particle tracking analysis (NTA) further demonstrated that the predominant diameter of the vesicles was 100 nm (Figure 2A), that are typical exosomes. Moreover, MGC803/DDP cells secreted significantly more exosomes than MGC803 cells (Figure 2B). By Western blot analysis, the exosomal specific markers (CD63 and CD81) were positive in the exosomes, whereas β‐tubulin was enriched in the whole cell lysates (Figure 2C). After that, PHK67 labelled MGC803 and MGC803/DDP exosomes (green fluorescence) were co‐cultured with MGC803 CM. As expected, green fluorescence was observed in exosomes treated MGC803 cells while no signal in PBS treated cells (Figure 2D). The uptake efficiency of exosomes by MGC803 cells escalated in a time‐dependent way and more than 80% cells were positive for fluorescence at 24 hours (Figure 2E). Thereby, we examined the effects of MGC803/DDP exosomes in DDP resistance in vivo and in vitro. Proliferation assay showed that MGC803/DDP exosomes increased MGC803 cell viability and IC_50_ values compared with PBS or MGC803 exosomes (Figure 2F). In MGC803 cells abdominal tumorigenesis assay, MGC803/DDP exosomes accelerate tumour growth and dissemination under DDP therapy (Figure 2G‐I). Besides, the sphere formation capability (Figure 2J) and CSCs properties (Figure 2K) of MGC803 cells increased when co‐culturing with exosomes from MGC803/DDP rather than MGC803. These results indicated that the exosomes isolated MGC803/DDP contributed to disseminate DDP resistance and promote CSCs properties in recipient MGC803 cells.

### 3.3 MGC803/DDP‐derived exosomes enhance DDP resistance of MGC803 recipient cells via exosomal miR‐500a‐3p delivery in vitro and in vivo

**FIGURE 3:**
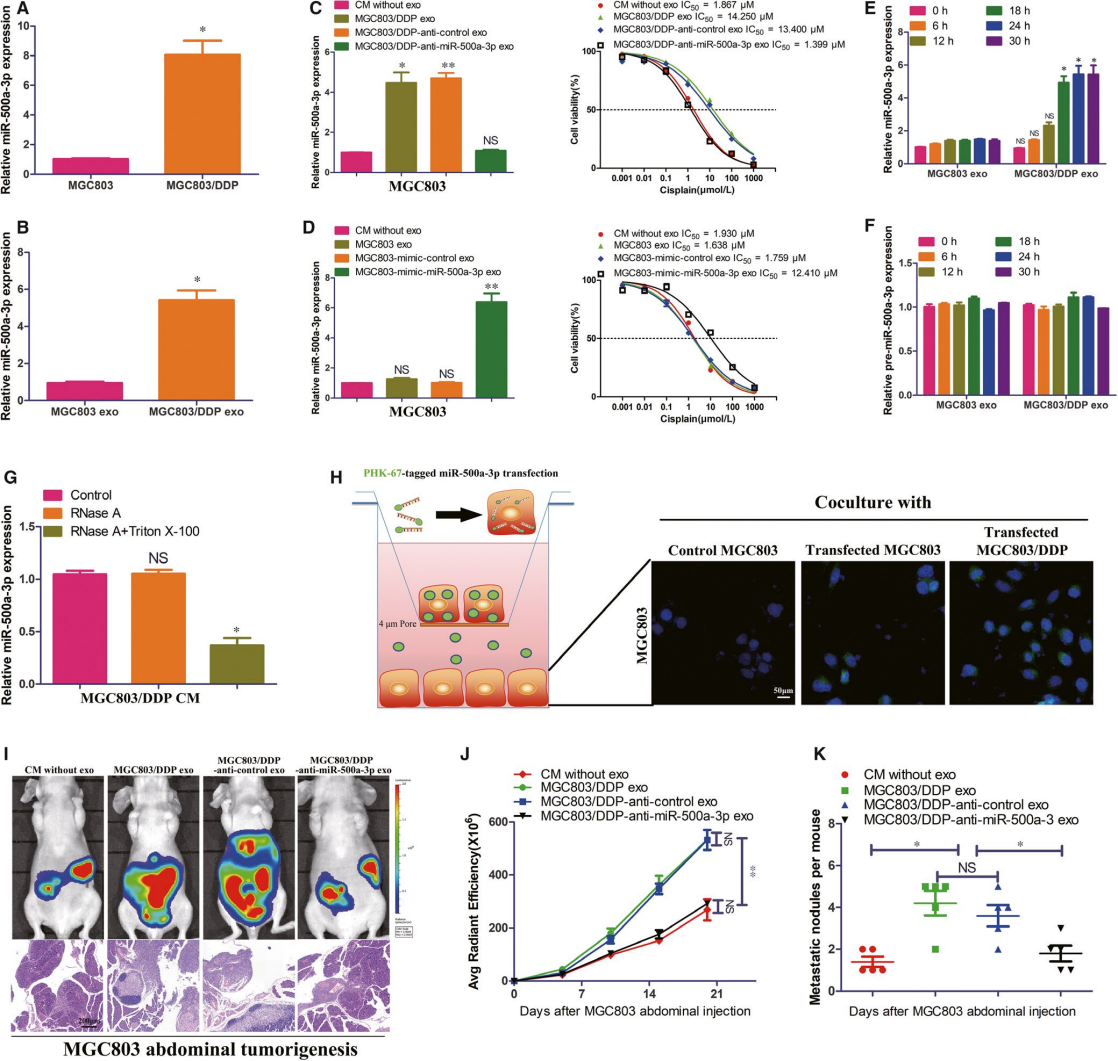
MGC803/DDP‐derived exosomes enhance DDP resistance of MGC803 recipient cells via exosome‐mediated delivery of miR‐500a‐3p in vitro and in vivo. A, B, Real‐time qRT‐PCR analysis of miR‐500a‐3p expression in MGC803, MGC803/DDP and their corresponding exosomes. C, D, Real‐time qRT‐PCR analysis of miR‐500a‐3p expression in MGC803 cells treated by indicated conditions and their corresponding proliferation assay analysis as well as IC_50_ value under DDP treatment. E, F, Real‐time qRT‐PCR analysis of miR‐500a‐3p or pre‐miR‐500a‐3p expression in MGC803 cells treated by MGC803 exosomes or MGC803/DDP exosomes at indicated time. G, Real‐time qRT‐PCR analysis of miR‐500a‐3p expression in the culture medium (CM) of MGC803/DDP after treatment with RNase (2 μg/mL) alone or combined with Triton X‐100 (0.1%) for 20 min. H, MGC803 or MGC803/DDP cells transfected with the PHK67‐miR‐500a‐3p mimic (green fluorescence) were placed in the upper chamber and coincubated with MGC803 cells seeded in the lower chamber in a transwell system with a 0.4 μm pore membrane. After coincubation for 30 h, MGC803 cells in the lower chamber were examined by the fluorescence microscope. I, Representative bioluminescent images and microscopy observations showed effects of CM without exosomes or indicated MGC803 cell‐derived exosomes on abdominal tumorigenesis under DDP treatment. J, K, Comparison of bioluminescent signals and abdominal metastatic nodules after indicated treatment, five nude mice in each group. *P < 0.05, **P < 0.01

To identify DDP resistance associated miRNAs in GC cells, we compared the miRNAs expression profile between MGC803/DDP and MGC803 cells by a miRNA microarray. The Figure S1 showed that a total of top 10 up‐regulated and 10 down‐regulated miRNAs between MGC803/DDP and MGC803 cells. Of these miRNAs, miR‐500a‐3p exhibited the most considerable degree of up‐regulation. Then, we validated the expression of miR‐500a‐3p in MCG803, MGC803/DDP and their secreted exosomes. As shown in Figure 3A,B, miR‐500a‐3p expression was significantly higher both in MGC803/DDP and their secreted exosomes by real‐time qRT‐PCR. We assumed that miR‐500a‐3p from MGC803/DDP exosomes may confer DDP resistance to recipient MGC803 cells by exosome. In the coincubation experiments, MGC803 intracellular miR‐500a‐3p levels were dramatically up‐regulated upon incubation with exosomes from MGC803/DDP with miR‐500a‐3p higher expression but not with exosomes from MGC803/DDP with miR‐500a‐3p knockdown by anti–miR‐500a‐3p transfection (Figure 3C). While incubation with exosomes from MGC803 with miR‐500a‐3p overexpression by mimic‐miR‐500a‐3p increased MGC803 intracellular miR‐500a‐3p level (Figure 3D). Functionally, MGC803 cells became insensitive to DDP when incubated with exosomes with higher miR‐500a‐3p, whereas miR‐500a‐3p down‐regulation in exosomes abolished this effect (Figure 3C,D). Moreover, the elevation of miR‐500a‐3p in recipient cells exhibited a time‐dependent manner after incubation with MGC803/DDP exosomes (Figure 3E). However, the level of pre‐ miR‐500a‐3p (precursor of miR‐500a‐3p) was not changed when incubating with MGC803/DDP exosomes (Figure 3F), suggesting the miR‐500a‐3p elevation in recipient cells was more likely a direct transfer by exosomes. Subsequently, we found miR‐500a‐3p expression in MGC803/DDP CM was little changed upon RNase A addition but significantly reduced when treated with RNase A + Triton X‐100 (Figure 3G), indicating that extracellular miR‐500a‐3p was mainly in the membrane. To visualize miR‐500a‐3p transfer, MGC803 and MGC803/DDP cells transiently transfected with PHK67‐tagged miR‐500a‐3p were co‐cultured with MGC803 cells for 30 hours in a transwell system, as depicted in Figure 3H. As a result, the green fluorescently labelled miR‐500a‐3p was observed in the lower chamber cells through confocal microscopy (Figure 3H), further suggesting that miR‐500a‐3p could be transferred by exosomes. In abdominal tumorigenesis model, MGC803/DDP exosomes promoted tumour growth and dissemination under DDP therapy but down‐regulating miR‐500a‐3p in MGC803/DDP exosomes could abolished its tumour promoting effect (Figure 3I‐K). These findings revealed that functional exosomal miR‐500a‐3p from DDP resistant GC cells could be transferred to recipient ones, which subsequently became resistant to DDP in vivo and in vitro.

### 3.4 MGC803/DDP‐derived exosomal miR‐500a‐3p confers DDP resistance in recipient MGC803 cells via inhibiting FBXW7

**FIGURE 4:**
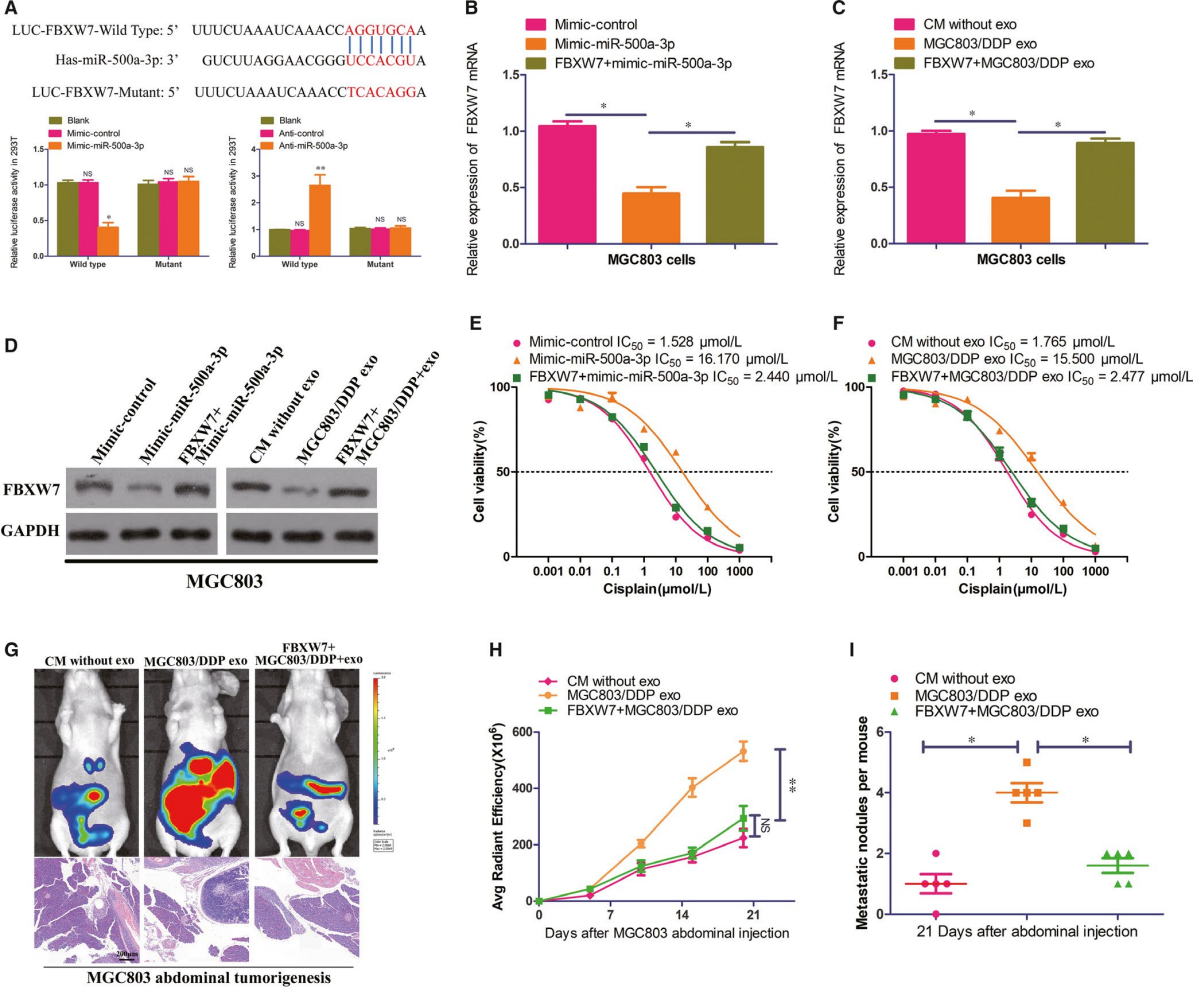
MGC803/DDP‐derived exosomal miR‐500a‐3p confers DDP resistance in recipient MGC803 cells via inhibiting FBXW7. A, Sequences of miR‐500a‐3p and the potential miR‐500a‐3p‐binding sites at the 3′UTR of FBXW7, including nucleotides mutated in FBXW7‐3′‐UTR. Seed sequences are marked. Also shown effects of Blank, mimic or anti–miR‐500a‐3p and corresponding control on the luciferase activity of FBXW7 3′UTR‐Wild‐Type and FBXW7 3′UTR‐Mutant by dual‐luciferase reported assay in 293T cells. B‐D, Expression of FBXW7 in MGC803 or FBXW7 overexpressed MGC803 cells transfected with mimic‐control or mimic‐miR‐500a‐3p by real‐time qRT‐PCR (B) and Western blot analysis (D). Expression of FBXW7 in MGC803 or FBXW7 overexpressed MGC803 cells co‐cultured with CM without exosomes or MGC803/DDP exosomes by real‐time qRT‐PCR (C) and Western blot analysis (D). E, Proliferation assay analysis and IC_50_ value in MGC803 or FBXW7 overexpressed MGC803 cells transfected with mimic‐control or mimic‐miR‐500a‐3p. F, Proliferation assay analysis and IC_50_ value in MGC803 or FBXW7 overexpressed MGC803 cells co‐cultured with CM without exosomes or MGC803/DDP exosomes. G, Representative bioluminescent images and microscopy observations showed effects of CM without exosomes, MGC803/DDP exosomes on abdominal tumorigenesis by MGC803 or FBXW7 overexpressed MGC803 cells abdominal tumorigenesis under DDP treatment. H, J, Comparison of bioluminescent signals and abdominal metastatic nodules after indicated treatment, five nude mice in each group. *P < 0.05 and **P < 0.01

By the publicly available algorithms TargetScan and mirDIP 4.1, we found that FBXW7 was the potential targets of miR‐500a‐3p with high predictive values. To verify the repression of miR‐500a‐3p on FBXW7, we constructed wide type and mutated FBXW7 3′‐UTR luciferase reporter according to TargetScan algorithm. Dual‐luciferase activity assay showed that the luciferase activity of FBXW7 with wild‐type 3′UTR was significantly inhibited in mimic‐miR‐500a‐3p transfected 293T, whereas anti–miR‐500a‐3p specifically abolished this suppression. Moreover, mutations in the miR‐500a‐3p binding seed region of the FBXW7 (LUC‐FBXW7‐Mutant) abrogated these above effects of mimic‐ or anti–miR‐500a‐3p transfection (Figure 4A). Moreover, both mRNA and protein expressions of FBXW7 were significantly decreased in MGC803 cells transfected with mimic‐miR‐500a‐3p or co‐cultured with MGC803/DDP exosomes while reintroduction of FBXW7 abolished the miR‐500a‐3p up‐regulation or MGC803/DDP exosomes induced FBXW7 decrease (Figure 4B‐D). To further elucidate the functional role of FBXW7 in miR‐500a‐3p mediated DDP resistance, we constructed FBXW7‐expressing plasmid. In proliferation assay, reintroduction of FBXW7 in MGC803 cells could reverse miR‐500a‐3p mediated DDP resistance. MGC803 abdominal tumorigenesis assay further revealed that reintroduction of FBXW7 suppressed tumour growth and dissemination under DDP therapy. Collectively, these results suggested that exosomal miR‐500a‐3p promoted DDP resistance in MGC803 cells through FBXW7 down‐regulation.

### 3.5 FBXW7 reversed the DDP resistance of exosomal miR‐500a‐3p by inhibiting CSCs properties

**FIGURE 5:**
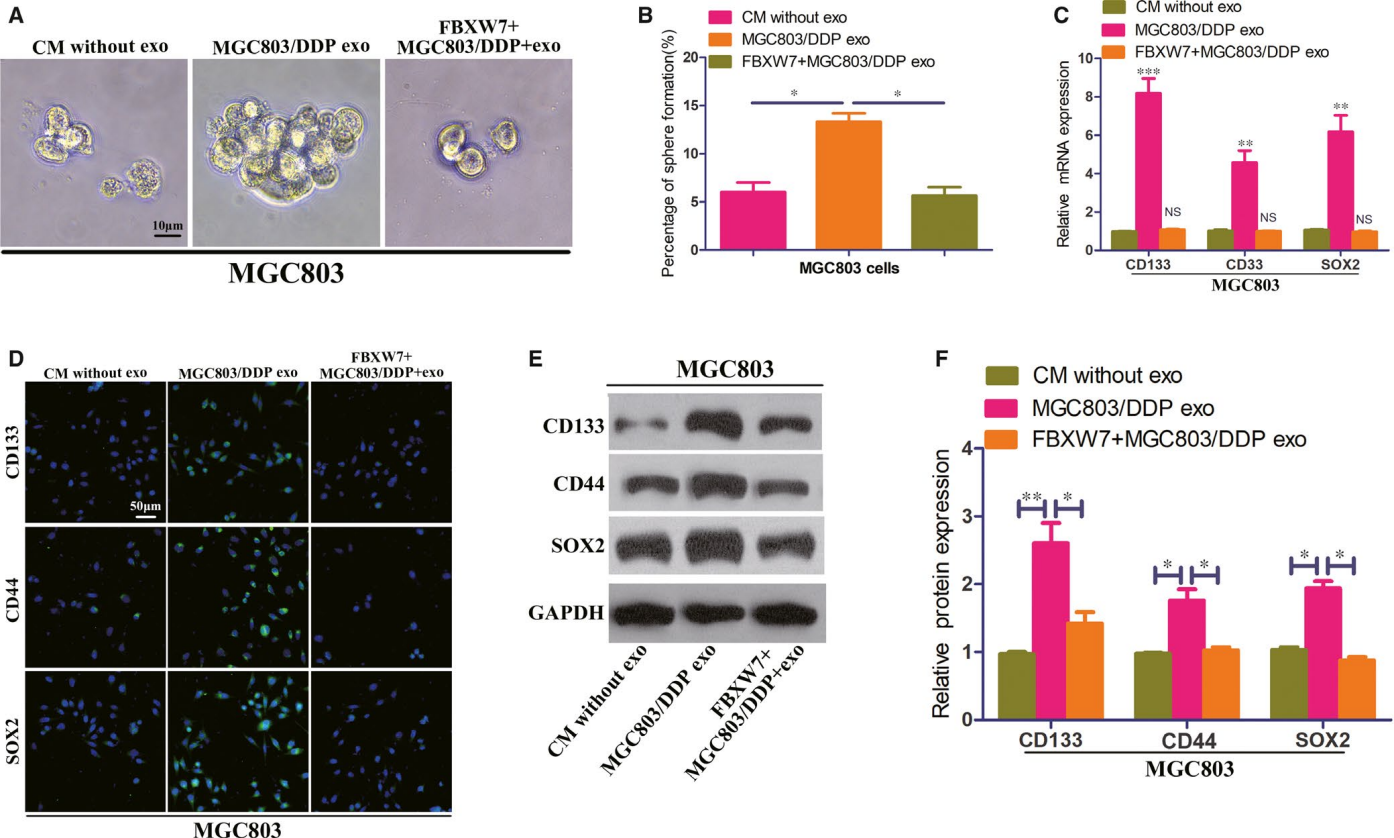
FBXW7 reverse the DDP resistance of exosomal miR‐500a‐3p by inhibiting CSCs properties in MGC803 cells. A, B, Effect of CM without exosomes, MGC803/DDP exosomes on sphere formation in MGC803 or FBXW7 overexpressed MGC803 cells. C, Relative mRNA expression of stemness markers CD133, CD44 and SOX2 in MGC803 or FBXW7 overexpressed MGC803 cells co‐cultured with CM without exosomes or MGC803/DDP exosomes. D‐F, Expression level of stemness markers in indicated GC cells by confocal microscopy (D) and Western blot (E, F). *P < 0.05, **P < 0.01 and ***P < 0.001

To study the mechanisms of FBXW7 in abrogating the DDP resistance induced by miR‐500a‐3p, we investigated the CSCs properties in GC. In sphere formation assay, MGC803/DDP exosomes induced more number and size of sphere formation were abrogated by FBXW7 overexpression (Figure 5A,B). Additionally, up‐regulation of cell stemness markers CD133, CD44 and SOX2 by MGC803/DDP exosomes could be inhibited by reintroduction of FBXW7 (Figure 5C‐F). Parallel results were also observed in MKN45 cells (Figure S2). These above data demonstrated that exosomal miR‐500a‐3p/ FBXW7 axis enhances DDP resistance in GC cells by CSCs properties activation.

### 3.6 Plasma exosomal miR‐500a‐3p is related to DDP resistance in III stage GC patients

**FIGURE 6:**
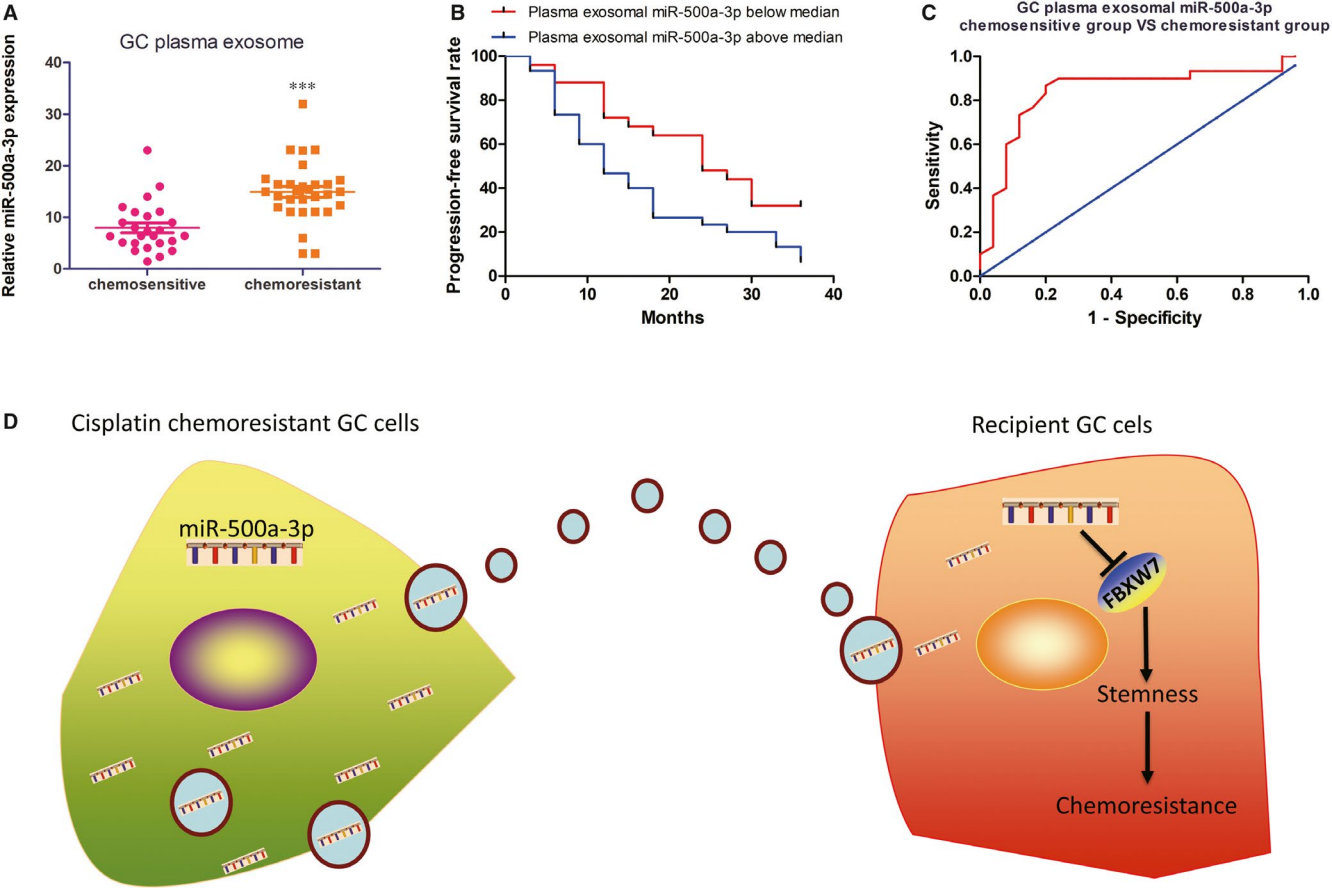
Plasma exosomal miR‐500a‐3p is related to DDP resistance in III stage GC patients. A, Plasma exosomal miR‐500a‐3p level was detected in III stage GC patients responding or not responding to DDP treatment by real‐time qRT‐PCR. B, Kaplan‐Meier analysis of 3 y progression‐free survival rate in III stage GC patients from high (n = 30) and low (n = 25) miR‐500a‐3p groups, according to the median exosomal miR‐500a‐3p level in pre‐gastrectomy plasma. C, ROC curve analysis of plasma exosomal miR‐500a‐3p expression for discriminating the DDP resistant group (n = 30) from the sensitive group (n = 25). AUC, area under the curve. D, Schematic diagram of the potential roles of exosomal miR‐500a‐3p in GC DDP resistance. Briefly, the DDP‐resistant GC cell‐secreted exosomes containing miR‐500a‐3p can be taken up by surrounding DDP sensitive GC cells and subsequently down‐regulate FBXW7 to enhance DDP resistance as well as CSCs properties of the recipient GC cells. ***P < 0.001

Clinically, we investigated the miR‐500a‐3p level in plasma exosomes and GC tissues of III stage GC patients who would receive DDP‐based chemotherapy. As presented in Figure 6A and Figure S3A, the miR‐500a‐3p levels were significantly higher in both plasma exosomes and GC tissues from DDP resistant patients than in those from DDP sensitive patients, while the FBXW7 expression was lower in DDP resistant patients compared with sensitive patients. These results further supported that FBXW7 was the downstream target of miR‐500a‐3p. Moreover, Kaplan‐Meier analysis revealed that high expression of exosomal miR‐500a‐3p levels in III stage GC patient plasma was negatively correlated with prognosis (Figure 6B). Importantly, receiver operating characteristic (ROC) curve analysis demonstrated that the ability to discriminate between the resistant and sensitive group with the plasma exosomal miR‐500a‐3p level was acceptably accurate (AUC = 0.843, Figure 6C). Above all, the plasma exosomal miR‐500a‐3p might be applied as the non‐invasive biomarker for DDP resistance in GC.

## 4 DISCUSSION

In spite of DDP‐based chemotherapy is still effective in a large number of malignancies, the emerge of DDP resistance is still an unavoidable difficulty for cancer patients,^21^, ^22^ especially in GC.^23^, ^24^, ^25^ Clinically, the overall 5‐year survival rate for GC patients who received DDP‐based chemotherapy after surgery remains dismal, while for late‐stage cases, DDP has shown little benefits because of dissatisfactory treatment efficiency, resulting in tumour progression and reduced prognosis.^5^ Therefore, figuring out the molecular mechanisms underlying DDP resistance may be of great assistance for improving GC patient outcome.^4^, ^26^, ^27^ In current study, the effects and mechanism of exosomal miR‐500a‐3p in DDP resistance were explored in GC cell. Our data suggested that miR‐500a‐3p abundance was elevated in DDP resistant GC cells and their secreted exosomes. Moreover, we found that exosomal miR‐500a‐3p could contribute to DDP resistance in recipient GC cells by down‐regulating FBXW7 expression via enhancing stemness cells properties.

There have been several reports showing that chemotherapy is capable to stimulate cancer cells to release more exosomes.^28^, ^29^ Lv et al^30^ reported that paclitaxel, irinotecan and carboplatin significantly increase the abundance of exosomes released from HepG2 (hepatocellular carcinoma cells). In breast cancer, Kreger et al^31^ found that, compared with those untreated MDA‐MB231 cells, the number of exosomes sheds by the MDA‐MB231 cells increased after paclitaxel treatment. Besides in vitro model, paclitaxel was reported to result in a higher amount release of exosomes in 4T1‐bearing mice and even in breast cancer patients, more exosomes were secreted after post‐neoadjuvant chemotherapy as compared with the basal levels.^32^, ^33^ Similar to above‐mentioned references, our study showed that DDP resistant cells (MGC803/DDP and MKN45/DDP) could release more exosomes than their parental ones. The higher exosomes release induced by chemotherapy is probably because of the cellular stress and damage resulted from chemotherapy. This process is resembling to how cells release damage‐associated molecular patterns (DAMPs).^34^, ^35^

Recently, miRNAs have been reported to be encapsulated in tumour‐derived exosomes to avoid degradation and subsequently those exosomal miRNAs would transfer to recipient cells to regulate genes expression, including angiogenesis, invasion and metastasis.^36^, ^37^ While for chemoresistance, those cells may release exosomal miRNAs into the extracellular environment and induce drug resistance to surrounding cells.^38^, ^39^ Exosomal miR‐196a derived from cancer‐associated fibroblasts result in head and neck cancer resistance to DDP.^38^ Exosomal miR‐126a have been reported to be involved in the doxorubicin resistance of lung cancer.^19^ Our results proved that exosomes from DDP resistant GC cells enhance recipient cells resistance to DDP by miR‐500a‐3p/FBXW7 pathway in vitro and in vivo.

MiR‐500a‐3p has been reported to be involved in the chemoresistance, invasion and migration via GSK‐3β and LY6K in different types of cancers.^40^, ^41^, ^42^ In this study, we found that miR‐500a‐3p was elevated in exosomes from DDP resistant GC cells and clinical up‐regulation of miR‐500a‐3p in exosomes from III stage GC patients' plasma correlated with DDP‐based chemoresistance and GC progression, which might be used as a non‐invasive predictor of chemotherapy in GC Patients. Furthermore, FBXW7 was identified as the target of miR‐500a‐3p in GC. FBXW7 (F‐box with 7 tandem WD40) is one of the crucial components of ubiquitin ligase that aids in the degradation of many oncoproteins via the ubiquitin‐proteasome system. FBXW7 is regarded as a potent tumour suppressor in different human cancers, as most of its target substrates can function as potential growth promoters.^43^ For instance, FBXW7 inactivation sensitized cancer cells to radiation or etoposide by stabilizing p53 to induce cell‐cycle arrest and apoptosis.^44^ While in GC, low expression of FBXW7 was observed in primary GC and contributed to the poor survival and minimal response to adjuvant therapy.^45^ Down‐regulation of FBXW7 by miR‐223 in GC cells promote proliferation, invasion and chemoresistance to trastuzumab in vitro.^46^, ^47^ Here, we found that overexpression of FBXW7 suppressed exosomal miR‐500a‐3p induced CSCs properties and thus reversed exosome mediated DDP resistance in GC.

In conclusion, we provide evidence that DDP resistance GG cells can secret miR‐500a‐3p enriched exosomes to promote stemness and DDP resistance by targeting FBXW7 in GC cells (Figure 6D). Moreover, exosomal miR‐500a‐3p is up‐regulated in the plasma of GC patients with DDP resistance, which thereby results in poor progression‐free prognosis. We assume that inhibiting exosomal miR‐500a‐3p could be used as a potential modality for the prediction and treatment of GC with chemoresistance.

## CONFLICT OF INTEREST

The authors declare they have no competing interests.

## AUTHOR CONTRIBUTIONS

**Pengpeng Liu:** Conceptualization (lead); Validation (lead); Writing‐original draft (lead); Writing‐review & editing (lead). **Hao Lin:** Data curation (equal); Formal analysis (equal); Investigation (equal); Supervision (equal). **Liang Zhang:** Data curation (equal); Investigation (equal); Resources (equal). **Caihua Zhang:** Data curation (equal); Validation (equal).

## ETHICAL APPROVAL

The study was approved by the medical ethics committee of XuZhou Central Hospital.

## CONSENT FOR PUBLICATION

We have received consent from individual patients who have participated in this study. The consent forms will be provided upon request.

## Supporting information

Supplementary Material

## Data Availability

The data used to support findings of the study are available from the corresponding author upon request.
