# Supplementary material for: Exosomal MiR‐500a‐3p promotes cisplatin resistance and stemness via negatively regulating FBXW7 in gastric cancer
Source: J Cell Mol Med. 2020 Jun 25;24(16):8930–41. doi: 10.1111/jcmm.15524 (PMC7417713; doi:10.1111/jcmm.15524)
Supplement: Supplementary file 1 — Supplementary Material [file JCMM-24-8930-s001.docx]

Supplement figure 1


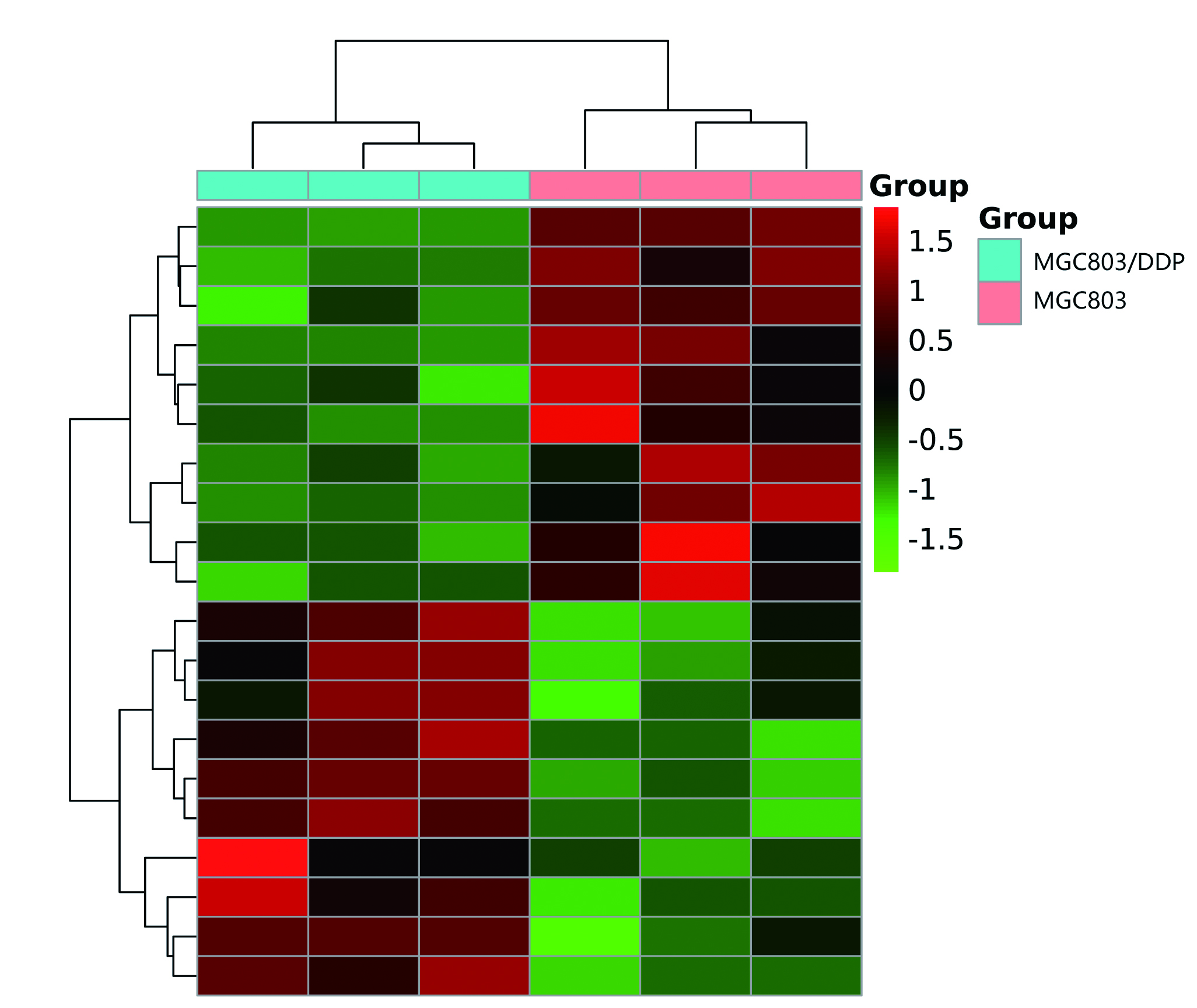


Supplement figure 2


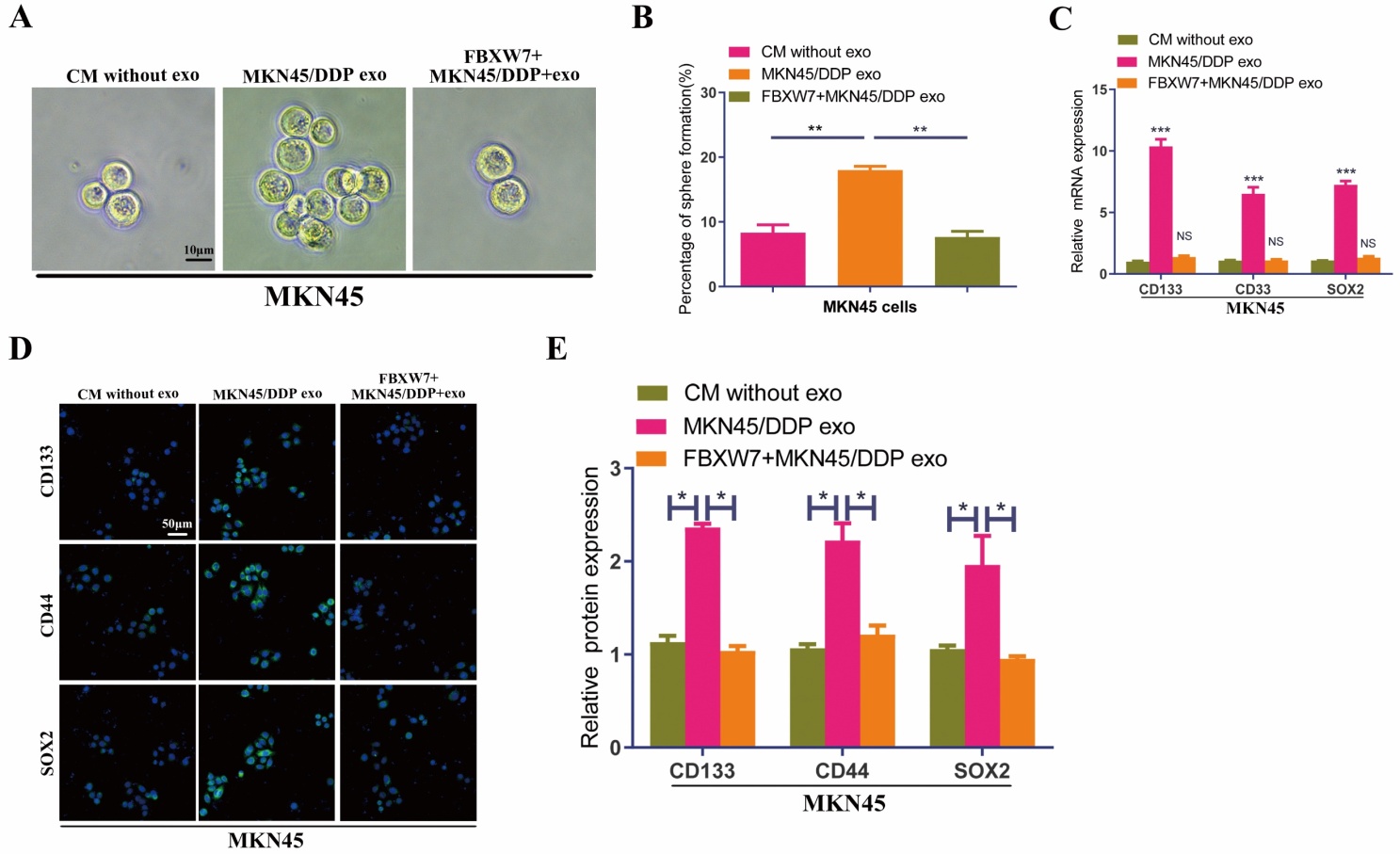


Supplement figure 3


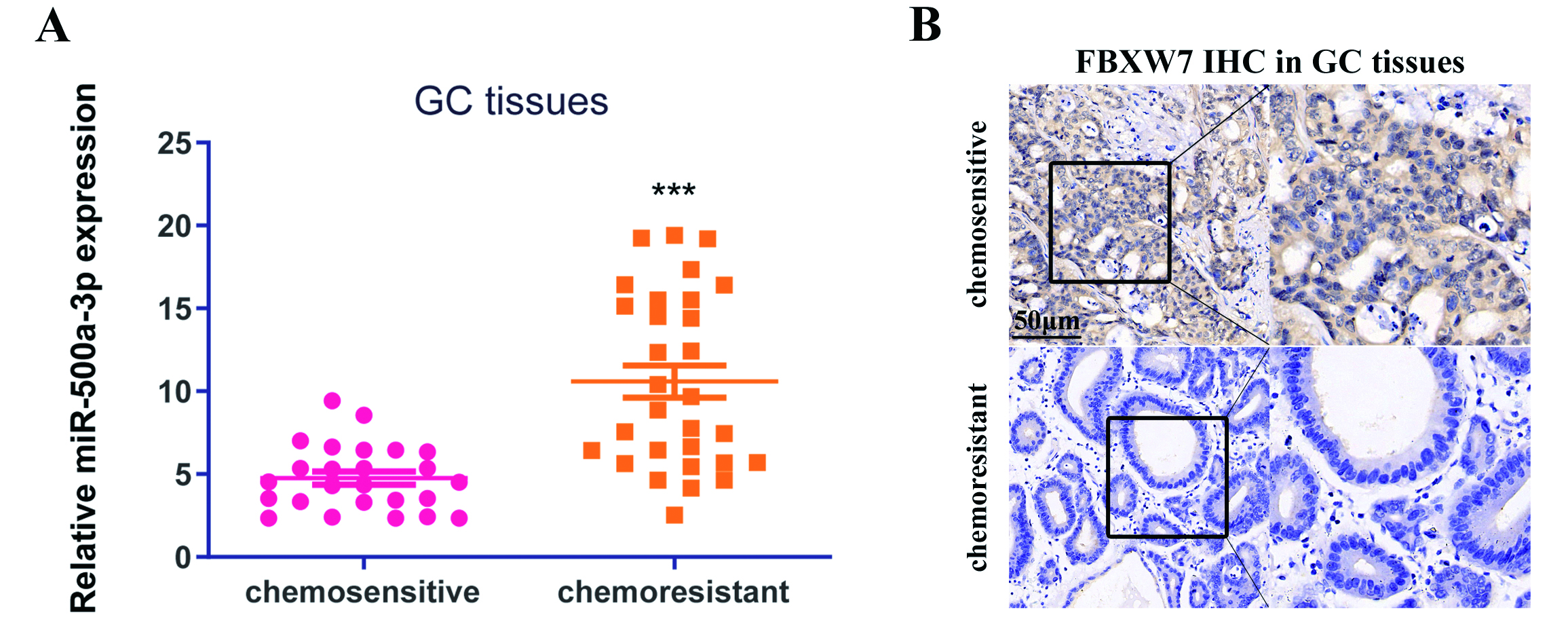


**Table S1.**

**The primer sequences used in real-time PCR**

| Gene | Primer sequence |
| --- | --- |
| miR-500a-3p | F: 5'-TTGAACCAAGGTTCGTAAATACCAA-3' |
| U6 | F: 5’-CTCGCTTCGGCAGCACA-3’ |
| FBXW7 | F: 5'-CGAACTCCAGTAGTATTGTGGACCT-3' |
|  | R: 5'-TTCTTTTCATTTTTGTTGTTTTTGTATAGA-3' |
| CD133 | F: 5'-AGTCGGAAACTGGCAGATAGC-3' |
|  | R: 5'-GGTAGTGTTGTACTGGGCCAAT -3' |
| CD44 | F: 5'-CTGCCGCTTTGCAGGTGTA-3' |
|  | R: 5'-CATTGTGGGCAAGGTGCTATT -3' |
| SOX2 | F: 5'-CTGGGTTGATCCTCGGACCT-3' |
|  | R:5’-CTGCGTAGTTGTGCTGATGT-3’ |
| GAPDH | F: 5’-TACTAGCGGTTTTACGGGCG-3' |
|  | R: 5’-TCGAACAGGAGGAGCAGAGAGCGA-3’ |

**Antibodies**

| Antibody | Assay | Product code |
| --- | --- | --- |
| CD63 | WB | Ab59479, Abcam, USA |
| CD81 | WB | Ab79559, Abcam, USA |
| β-tubulin | WB | Ab179511, Abcam, USA |
| FBXW7 | WB, IHC | Ab109617, Abcam, USA |
| GAPDH | WB | 10494-1-AP, Proteintech, USA |
| CD133 | WB, IF | 18470-1-AP, Proteintech, USA |
| CD44 | WB, IF | 15675-1-AP, Proteintech, USA |
| SOX2 | WB, IF | Ab93689, Abcam, USA |
